# Erythema Annulare Centrifugum: A Rare Skin Finding of Autoimmune Hepatitis

**DOI:** 10.4021/gr2010.04.181w

**Published:** 2010-03-20

**Authors:** Cem Aygun, Orhan Kocaman, Yesim Gurbuz, Altay Celebi, Omer Senturk, Sadettin Hulagu

**Affiliations:** a Department of Gastroenterology, Kocaeli University Medical Faculty, Turkey; b Department of Pathology, Kocaeli University Medical Faculty, Turkey

**Keywords:** Erythema annulare centrifugum, Autoimmune hepatitis

## Abstract

Erythema annulare centrifugum is characterized by dermal perivascular lymphocytic infiltrates. It is often associated with infections, autoimmune or neoplastic diseases but in most cases the cause is unexplained. A case of erythema annulare centrifugum related to autoimmune hepatitis in a 24-year-old woman is described in this case report. Clinical response of the autoimmune hepatitis to a combination therapy with corticosteroids and azothiopurine was achieved. Although partially regressed for the first 12 months of theraphy, the skin lesions did not disappear completely. However, after 18 months of continious treatment there was no skin lesion.

## Introduction

Erythema annulare centrifugum (EAC) is characterized by dense perivascular lymphocytic infiltrate in dermis. It is often associated with various conditions including infectious, autoimmune or neoplastic diseases. But still in most cases of EAC, the cause remains unexplained. EAC is generally classified into a superficial and a deep type, although neither type is associated consistently with any other systemic disease [1]. The deep form is non-pruritic and non-scaly with firm borders and the superficial form is scaly and pruritic with non-indurated borders. The most distinctive feature of both type of EAC is dermal perivascular lymphocytic infiltrate [2].

Autoimmune hepatitis (AIH) is a serious and progressive inflammation of liver. Women are affected more than men and the cause is unknown. It reflects a complex interaction between foreign antigens as triggering factors and autoantigens as host factors, where in the end self tolerance is lost. Underlying immunopathic process may target other organs but generally specific skin findings are not available or sufficient to support the probable diagnosis of AIH. In this case report we present an AIH patient with associated skin lesions diagnosed as EAC.

## Case Report

**Figure 1:**
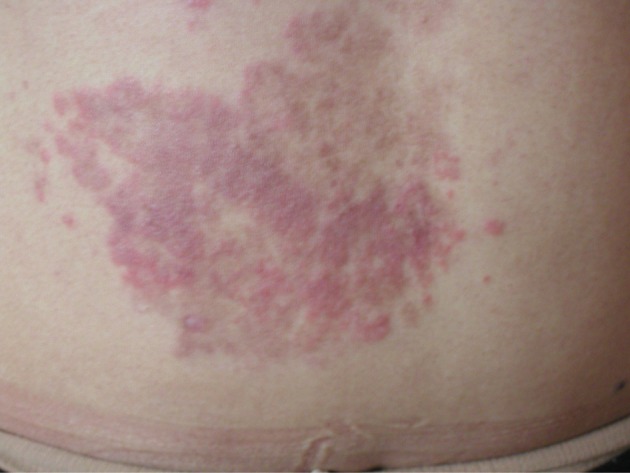
Erythematous lesion on the abdominal wall of patient. Skin changes started over the shoulder region initially, they were intermittent and not cosmetically disturbing, but after 2 months with the onset of overt autoimmune hepatitis, a larger and cosmetically disturbing EAC lesion developed.

A 24-year-old female presented with symptoms of upper abdominal discomfort, anorexia, jaundice and mild pruritis. She had fatigue for more than a month and jaundice was noticed for one week. The stools were intermittently pale with darkened urine for the last week. She also had an intermittent erythematous non-pruritic skin rash over the shoulder region for the past two months and after 2 weeks of jaundice a new-onset, larger annular skin rash on the abdominal wall became evident. On examination, the skin over the abdomen showed non-scaly annular erythematous plaques with firm margins (Fig. 1). Abdominal examination revealed no hepatosplenomegaly and no stigmata of chronic liver disease.

**Figure 2:**
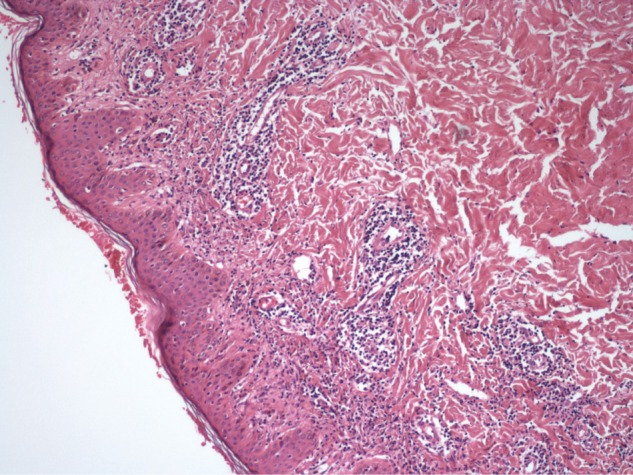
Histopathologic examination of the lesion showed diffuse perivascular infiltrate in the dermis (Haematoxylin & Eosin, x100).

Laboratory tests showed that serum aminotransferases (ALT,1202 IU/L; AST, 760 IU/L), bilirubin levels (total, 13.1 mg/dl; direct, 10.3 mg/dl) and alkaline phosphatase (ALP, 204 IU/L) were elevated. Serum prothrombin time was normal but there was reversal of albumin/globulin (A/G) ratio (albumin, 4.02 g/dl; globulin, 4.33 g/dl). Abdominal ultrasound revealed normal liver size with normal echo texture with no evidence of portal hypertension. Liver biopsy procedure was performed and showed lymphoplasmocytic infiltrate with piecemeal necrosis. The portal tract was expanded by mononuclear infiltrate, the limiting plate was disrupted and the inflammation extended to the acinus, suggesting interface hepatitis. The patient was negative for HBsAg, anti-HBs Ab, anti- HAV IgM, anti-HCV Ab and HCV RNA serology. Serum ferritin, ceruloplasmin levels were normal and there was no alpha-1 anti-trypsin deficiency. For immunoserologic markers, antinuclear antibodies (ANA) was positive (1:320) while smooth muscle antibodies (SMA) and antimitochondrial antibodies (AMA) were negative. Skin biopsy revealed a marked diffuse perivascular lymphocytic infiltrate with a tightly cuffed ‘coat-sleeve-like’ pattern in mid and deep dermis (Fig. 2).

The patient was diagnosed as a case of type-1 AIH associated with EAC, and started on prednisolone (60 mg/day, PO) immediately. During the initial follow-up although fatigue, jaundice and pruritis completely recovered, the erythematous rash on abdominal wall showed no change. The serum aminotrans­ferases (ALT and AST were 23 and 15 IU/L, respectively), biluribin levels (total 1.79 mg/dl, direct 1.28mg/dl) and A:G ratio normalized after 3 months of therapy. With clinical and biochemical remission glucocorticoid theraphy was titrated to the lowest level and discontinued. Meanwhile azothiopurine theraphy (2 mg/kg, PO) as a maintenance regimen was started. The skin rash on the abdominal wall persisted but partially regressed after 12 months of follow-up; the liver function test remained normal and immunosupression was continued with azathiopurine. After 18 months of follow-up there was no skin lesion with stable liver function tests.

## Discussion

EAC is generally thought to represent a cutaneous hypersensitivity reaction to underlying conditions such as infection by dermatophytes, bacteria and viruses, malignancy, and immunological disorders [3, 4]. The exact cause of EAC is not clear yet. AIH as an immunologic disease presents with hepatocellular inflammation and its cause is also unknown. Concurrent immunologic diseases are common in AIH. These involve diverse organ systems, most frequently the thyroid but not so frequently the skin [5]. Associated skin conditions include allergic capillaritis, erythema, LE type changes and purpura. Endocrinologic changes associated with AIH may also be responsible for skin changes such as acne, hirsutism, cutaneous stria or sometimes cushingoid appearance [6].

In this report, we presented a case of an AIH associated with a rare skin lesion diagnosed as EAC. Skin changes appeared two months before the clinical presentation of hepatitis initially over the shoulders and a larger new annular lesion developed on the abdominal wall after the onset of overt hepatitis. During the initial follow-up abdominal lesion persisted over a period of twelve months (with only partial regression), despite the symptoms and laboratory findings of liver disease were resolved with immunosuppressive theraphy used. Intermittant skin rashes around shoulder region, on the other hand disappeared shortly after the treatment with a better response.

EAC has already been reported to a wide variety of etiologic factors, including infections by bacteria, viruses, fungi, parasites, malignant neoplasms ranging from carcinomas to Hodgkin disease and other hematological malignancies, drugs such as antimalarials, cimetidine, aldactone, gold, and amitriptyline, sarcoidosis, Graves’ disease nephritis, cholelithiasis, and pregnancy [1-4]. Recently skin rashes diagnosed as EAC in a pediatric autoimmune hepatitis case has also been reported. The child was a 13-month-old boy, diagnosed as AIH with skin changes over the trunk, buttocks, thighs, face and arms which showed scaly pruritic annular erythematous plaques with non-indurated margins [7]. With our case, we were able to report another presentation of an AIH associated with EAC as a skin finding. To our present knowledge, this is the first reported association of EAC and AIH in an adult patient.

EAC is mainly categorized into deep and superficial variants [8]. Clinical presentation of a deep form of EAC is an annular lesion with nonscaling and indurated edges. Histopathologically, a dense perivascular infiltrate is present in the middle and lower portions of the dermis. On the other hand, diagnosis of superficial form is established by less induration and this form as a characteristic feature shows scaling along a ring-shaped border. Additionally parakeratosis and spongiosis may be found in superficial form EAC’s histopahology. Regarding clinical findings of the superficial and the deep type of EAC, it has been generally stated that the superficial variant differs only by the presence of a characteristic trailing scale and a delicate annular rim of scale that trails behind the advancing edge of erythema. The skin lesion over the abdominal wall in our patient was consistent with the deep form of EAC, both clinically and histopathologically. Absence of scaling was the most important clinical difference indicating the deep type of EAC in our case.

Because of various associated conditions, EAC is currently considered to be a distinctive hypersensitivity reaction that may be initiated by many different antigens. Effector T-lymphocytes recognize antigen presented on the surface of hepatocyte by autologous HLA molecules. An interaction between antigenic peptide and the HLA molecule is necessary. This assumption is supported by a paralel course of annular skin lesions and associated diseases in many cases reported in the literature. Molecular mimicry of a foreign antigen and self antigen is the most common explanation for the loss of self-tolerance, but there is no autoimmune disease in which this mechanism has been clearly established yet [9]. Genetic factors may influence autoantigen presentation and helper T cell recognition. In our case also, common antigen-antibody related immunological mechanisms might be involved in the development of both AIH and EAC, and these two distinct clinical pictures might probably be the stages of the same pathogenic entity.

In summary, new clinical features of AIH such as EAC continue to be recognized because the the underlying immunopathic process of the disease may target any organ (as well as skin) as autoantigen. Skin changes including EAC can be associated with AIH, can show rapid progression with disease activity and can resolve completely with successful treatment.
